# Engineering combinatorial and dynamic decoders using synthetic immediate-early genes

**DOI:** 10.1038/s42003-020-01171-1

**Published:** 2020-08-13

**Authors:** Pavithran T. Ravindran, Maxwell Z. Wilson, Siddhartha G. Jena, Jared E. Toettcher

**Affiliations:** 1 Department of Chemical and Biological Engineering, Princeton University, Princeton, NJ 08544 USA; 2 Department of Molecular Biology, Princeton University, Princeton, NJ 08544 USA; 3 Department of Molecular, Cellular, and Developmental, Biology, University of California, Santa Barbara, CA USA

**Keywords:** Genetic circuit engineering, Growth factor signalling, Synthetic biology

## Abstract

Many cell- and tissue-level functions are coordinated by intracellular signaling pathways that trigger the expression of context-specific target genes. Yet the input–output relationships that link pathways to the genes they activate are incompletely understood. Mapping the pathway-decoding logic of natural target genes could also provide a basis for engineering novel signal-decoding circuits. Here we report the construction of synthetic immediate-early genes (SynIEGs), target genes of Erk signaling that implement complex, user-defined regulation and can be monitored by using live-cell biosensors to track their transcription and translation. We demonstrate the power of this approach by confirming Erk duration-sensing by *FOS*, elucidating how the *BTG2* gene is differentially regulated by external stimuli, and designing a synthetic immediate-early gene that selectively responds to the combination of growth factor and DNA damage stimuli. SynIEGs pave the way toward engineering molecular circuits that decode signaling dynamics and combinations across a broad range of cellular contexts.

## Abstract (web summary)

Ravindran et al. report the construction of synthetic immediate-early genes (SynIEGs), target genes of the Erk signaling pathway. SynIEGs implement user-defined regulation while tracking transcription and translation. This study underscores post-transcriptional regulation in signal decoding that may be masked by analyses of RNA abundance alone.

## Introduction

**Fig. 1:**
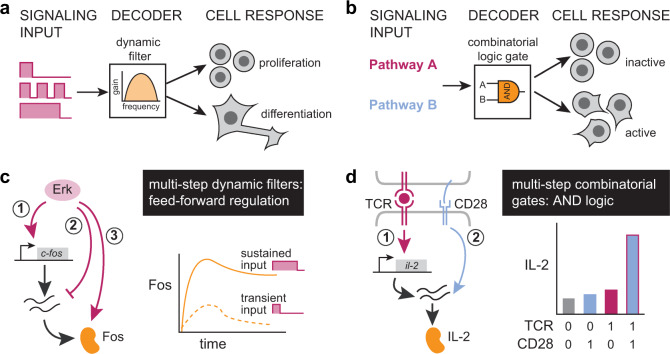
Cells use multi-step regulation to interpret dynamic and combinatorial signaling inputs. **a** Target gene induction may depend on the dynamics of signaling pathway activation, such as the duration, frequency, or area under the curve of pathway activity. In such cases, the signal-decoding circuitry may be thought of as a dynamic filter. **b** Target gene induction may also depend on the combination of pathways that are activated, such that signal decoding may be thought of as implementing a logic gate. **c** The induction of Fos protein is a canonical example of dynamic decoding, where sustained but not transient pulse of Erk results in protein accumulation. Erk-mediated regulation of *FOS* transcription, fos mRNA stability, and Fos protein stability is thought to mediate this response. **d** IL-2 induction by T cell stimulation and co-stimulation is thought to occur via combinatorial control. Neither TCR nor CD28 alone are sufficient for protein output but the two together allow for accumulation of IL-2 protein through a multi-step circuit.

In mammals, relatively few intracellular pathways integrate information from a huge range of sources, including neighboring cells and the physical environment. The resulting activity of signaling pathways can have many consequences, but chief among these is the induction of target genes that constitute a cell’s decision to proliferate, differentiate, or adopt an altered functional state. Efforts to systematically map signaling responses have revealed that a single external stimulus often activates many intracellular pathways, and that each pathway’s activity state can vary dynamically over time^1–4^. These observations suggest that cells might use both combinatorial strategies (e.g., gene expression triggered only in response to pathways A and B) and dynamic strategies (e.g., gene expression triggered by sustained but not transient activity in pathway A) to connect the cell’s overall signaling state to particular responses. A growing number of cell fates are thought to be selectively triggered by certain signaling dynamics, thereby functioning as analog filters (Fig. 1a)^5–7^, whereas others may act as digital logic gates by responding only to certain pathway combinations (Fig. 1b)^8^. Yet our understanding of how combinatorial and dynamic decoding are achieved is still limited.

A central challenge is that the relationship between signaling and target gene activation is complex, with multiple regulatory links acting at different steps along the central dogma, an architecture we will call “multi-step regulation.” One canonical example of multi-step regulation is found in *FOS* gene induction by the Ras/Erk pathway. Erk signaling first triggers transcription of fos mRNA; then, within 30–60 min, fos mRNA is degraded through the Erk-induced expression of Zfp36; finally, the Fos protein is stabilized by Erk phosphorylation^9–12^. Together, these interactions are thought to form a circuit that selectively responds only when the duration of Erk activity is above a threshold (Fig. 1c). Multi-step regulation can also provide combinatorial control when sequential steps in gene expression are gated by distinct signaling pathways. For instance, in T cells, engagement of the T cell receptor (TCR) leads to il-2 transcription, but maximal IL-2 protein secretion requires CD28-dependent signaling acting at post-transcriptional steps that are still poorly defined (Fig. 1d), resulting in AND-gate logic where both TCR and CD28 engagement are required for a strong cytokine response^13^. In both cases, the complexity of multiple nested regulatory links has made it challenging to define the essential set of interactions needed to implement a specific filtering or gating function. A more complete understanding of multi-step regulation would also enable the design of synthetic decoding modules: gene circuits that selectively respond to novel stimulus combinations or dynamics.

Motivated by these challenges, we set out to establish a general framework for constructing synthetic signaling-responsive target genes that can be used to implement user-defined, multi-step regulatory interactions. We decided to focus specifically on creating and characterizing synthetic immediate-early genes (SynIEGs), a class of fast-responding genes that are induced by a variety of stimuli including Ras/Erk signaling. We found that SynIEGs faithfully recapitulate the dynamics of immediate-early gene induction: SynIEG transcriptional kinetics closely match their endogenous counterparts, and a *FOS*-based SynIEG exhibits dynamic filtering of Erk signaling inputs. We also use the SynIEG platform to define additional regulatory links, revealing an essential role for the *BTG2* 3′ UTR and the microRNA miR-21 in suppressing Btg2 protein translation. Finally, we use regulatory elements from the *FOS* and *BTG2* genomic loci to engineer a SynIEG with a decoding function distinct from both its parent immediate-early genes: synergistic activation by the combination of growth factor and DNA damage stimuli. Synthetic signaling-responsive target genes thus enable a quantitative, systems-level understanding of the interface between signaling pathways and gene expression, opening the door to engineering pathway decoders for controlling complex cell fates.

## Results

### Developing SynIEGs for monitoring target gene induction

**Fig. 2:**
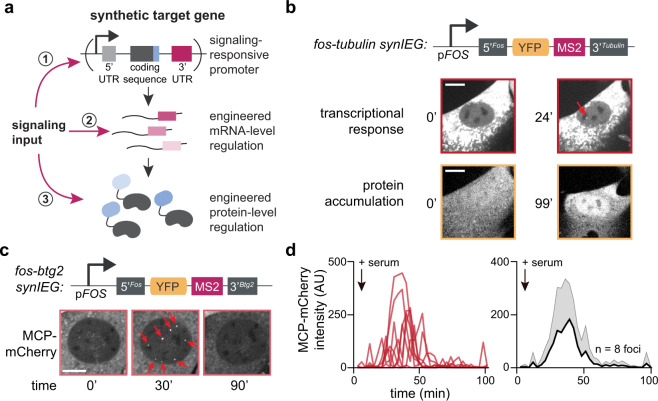
Development of synthetic Ras/Erk target genes that recapitulate endogenous transcriptional kinetics. **a** Schematic overview of synthetic target genes incorporating multi-step regulation. Signaling inputs can act at the transcriptional level through promoter/enhancer regulation (1), the mRNA level through UTR-based regulation (2), and at the protein level through signaling-responsive domains or linear motifs (3). **b** Design of synthetic immediate-early genes (SynIEGs). The Erk-responsive FOS promoter (*pFOS*) drives the expression of yellow fluorescent protein (YFP) followed by 24 MS2 stem-loops, which allow for the visualization of protein accumulation and transcription, respectively, in live cells. mRNA UTR elements and protein degrons can be added to modulate mRNA and protein stability. Representative images at different timepoints of a single cell expressing a SynIEG containing the *FOS* 5′ UTR and *TUBULIN* 3′ UTR before and after serum stimulation for both transcription and translation. Transcriptional focus of MCP-mCherry localization is denoted with a red arrow. Data from additional cells are shown in Supplementary Fig. 1. Scale bar is for 10 μm. **c** Images of an NIH3T3 nuclei after induction of transcription of SynIEG contain *FOS* 5′ UTR and *BTG2* 3′ UTR at multiple timepoints after serum stimulation. Transcriptional foci are denoted using red arrows. Scale bar is for 10 μm. **d** Quantification of the eight transcriptional foci in the cell shown in **c**, showing individual traces (left) their mean + SD (right). Transcriptional responses from 10 additional cells are shown in Supplementary Fig. 3.

Our strategy for constructing synthetic target genes is based on extensive classical studies using reporter gene assays to investigate the function of enhancers, promoters, and RNA/protein elements^14–17^. In our case, a synthetic target gene cassette must meet two complementary goals. First, a SynIEG must be able to easily modified and introduced in order to implement different forms of regulation at the mRNA or protein level. Each SynIEG thus combines a signaling-responsive promoter, 5′ and 3′ mRNA regulatory sequences, and protein-coding sequences into a plasmid that can be targeted for genomic integration (Fig. 2a, Supplementary Note 1), and in the following section we verify that these randomly integrated genes exhibited responses that matched their endogenous counterparts (Fig. 2, Supplementary Figs. 1–4). Second, a quantitative understanding of signal decoding requires the ability to monitor dynamic responses at both the mRNA and protein levels in single cells over time, dual capabilities that are rarely afforded in classic gene reporter assays^14–17^. Building off of the recent rapid development of live-cell transcriptional and translational reporters^18–20^ and a strategy we recently developed for endogenous target genes^21^, we included a YFP-24xMS2 tag in each SynIEG. In this system, instantaneous transcription can be visualized as a bright nuclear spot when the nascent MS2 RNA loops are bound to the fluorescent RNA-binding protein MCP-mCherry, and protein accumulation can be monitored by YFP fluorescence. As a first test case, we combined a 2-kb region upstream of the *FOS* transcriptional start site that contains its promoter and canonical upstream regulatory elements^22,23^, the *FOS* 5′UTR, the coding sequence for monomeric super-folder YFP (msfYFP), 24xMS2 RNA stem–loops, and the *TUBA1B* 3′UTR in a single lentiviral vector, which we named *fos-tubulin* for its 5′ and 3′ elements, respectively (Fig. 2b), and introduced it into a clonal NIH3T3 cell line already expressing MCP-mCherry and H2B-iRFP as a nuclear marker (the “chassis” cell line; see “Methods”)^21^.

We first tested whether or not expression of the *fos-tubulin* synthetic immediate-early gene (SynIEG) could be induced by signaling stimuli after either lentiviral transduction or transient transfection, but found that neither strategy was promising. Transient transfection with a *fos-tubulin* plasmid drove YFP expression even in the absence of IEG-activating stimuli such as serum (Supplementary Fig. 1a, b). Conversely, cells transduced with a *fos-tubulin* lentiviral vector failed to robustly induce YFP expression even after serum stimulation (Supplementary Fig. 1c). Based on prior reports that lentivirally integrated transgenes are often substrates for epigenetic silencing^24^, we reasoned that treatment with the histone deacetylase inhibitor trichostatin A (TSA) might restore serum responsiveness to a lentiviral *fos-tubulin* cell line. Indeed, cells pre-treated with TSA for 12 h exhibited an increase in YFP fluorescence upon serum stimulation, whereas cells treated with either TSA or serum alone showed no change in YFP levels (Supplementary Fig. 1c, d). While these data indicate that lentivirally transduced SynIEGs can be reactivated from a silent state, the large-scale chromatin remodeling caused by TSA treatment made this an undesirable strategy for implementing SynIEGs.

As an alternative method of synthetic gene delivery, we tested integration using the PiggyBAC transposase, which randomly inserts DNA sequences flanked by ~300-bp targeting sequences into the host genome^25^. We co-transfected “chassis” NIH3T3 cells with plasmids encoding the PiggyBAC transposase enzyme and the *fos-tubulin* SynIEG flanked by PiggyBAC transposable elements, and sorted clonal cell lines that stably integrated the SynIEG (Fig. 2b). We observed that *fos-tubulin* cells stimulated with serum exhibited bright MCP-labeled transcriptional foci and increased YFP fluorescence levels over time, consistent with an Erk-stimulated IEG response (Fig. 2b, Supplementary Fig. 1e, f). Unlike our lentiviral constructs, silencing was not observed and cells maintained serum responsiveness even months after cell line generation (Supplementary Fig. 1g). These results confirm that PiggyBAC transposase-based delivery enables the stable integration of complex, signaling-responsive synthetic target genes for interrogating mRNA- and protein-level responses.

How well do SynIEGs recapitulate the dynamics of endogenous immediate-early gene activation? To address this question, we monitored transcription at individual genomic loci using the MS2/MCP system built into each SynIEG. In this system, the transcription rate of each genomic integration site can be tracked over time based on the intensity of individual fluorescent MCP foci in the nucleus (Supplementary Fig. 2**)**. We generated a clonal cell line in which a *fos-btg2* SynIEG (containing the *FOS* 5′ regulatory sequence and *BTG2* 3′ UTR) was integrated at multiple genomic loci. We chose the *BTG2* 3′ UTR based on our prior work demonstrating it produces exceptionally bright transcriptional foci, likely due to its length relative to other immediate-early 3′UTRs^21^. Upon serum stimulation, *fos-btg2* cells exhibited ~8 bright transcriptional foci, corresponding to transcription from distinct PiggyBAC integration sites (Fig. 2c). Focus intensity reached a maximum intensity roughly 30 min after stimulation and then adapted back to baseline within 90 min (Fig. 2d, Supplementary Movie 1). Transcriptional kinetics were strikingly similar between distinct foci within the same cell, as well as between cells in the same cell line, indicating that SynIEGs are modular entities that can trigger similar responses regardless of genomic integration site (Supplementary Movie 1, Fig. 2d, Supplementary Fig. 3).

We next set out to compare the kinetics of serum-stimulated SynIEG transcription with their endogenous IEG counterparts. We had previously established derivatives of the “chassis” NIH3T3 cell line where MS2 stem–loops were integrated at the endogenous *FOS* and *BTG2* loci, providing an ideal test-bed for comparison^21^. We stimulated *fos-btg2* SynIEG cells and these endogenously-tagged *FOS* and *BTG2* cell lines with 10% serum (Supplementary Fig. 4a), and quantified both the kinetics of transcriptional activation and subsequent adaptation to the baseline state (Supplementary Fig. 4b). The transcriptional dynamics of the *fos-btg2* SynIEG were similar to those of the endogenous *FOS* genomic locus, consistent with the view that bursting kinetics are solely controlled by the proximal *FOS* enhancer–promoter sequence in our synthetic gene construct (Supplementary Fig. 4c). SynIEGs also recapitulated many prior observations about immediate-early gene regulation^21^: the transient pulse of SynIEG transcription was converted to a sustained transcriptional response by co-treatment with the protein synthesis inhibitor cycloheximide (Supplementary Fig. 4d, e), and serum-induced transcription was immediately blocked by pharmacological inhibition of the MAPK pathway (Supplementary Fig. 4f). Taken together, these data indicate that transposase-integrated SynIEGs are highly sensitive to upstream MAPK pathway signaling and suggest that SynIEGs quantitatively reflect the dynamics of gene expression of their endogenous IEG counterparts.

### SynIEGs recapitulate dynamic decoding by *FOS*

**Fig. 3:**
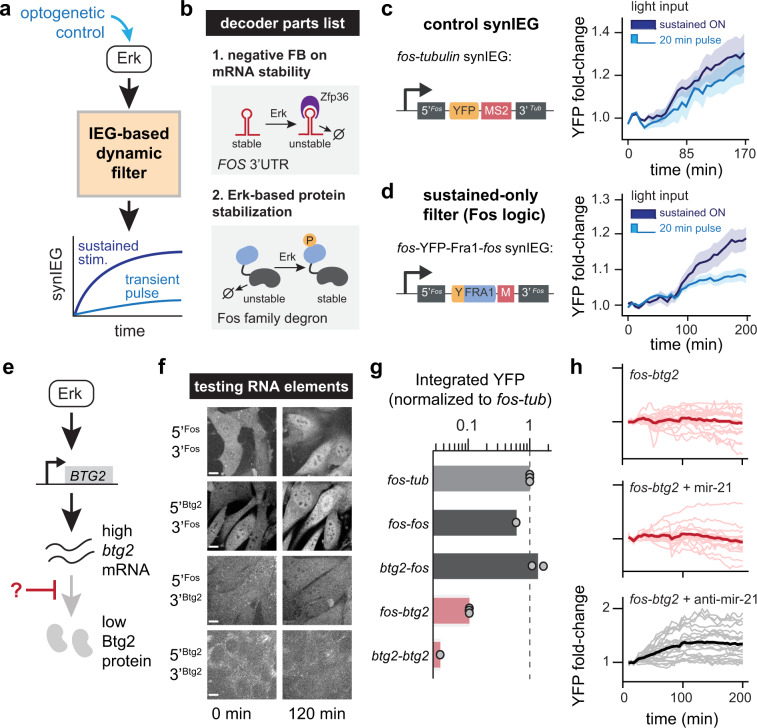
SynIEGs can be used to implement dynamic and combinatorial decoding circuits. **a** Schematic of platform to test whether a synthetic immediate-early gene regulatory circuit could recapitulate dynamic filtering by the *FOS* immediate-early gene using the OptoSOS optogenetic system for applying dynamic stimuli (see Supplementary Fig. 5). **b** Parts list of the sustained-only dynamic filter. The *FOS* 3′ UTR is subject to degradation by a second Erk-induced immediate-early gene, Zfp36 (upper panel). Protein levels are stabilized by Erk phosphorylation of a Fos-family degron sequence (lower panel). **c**, **d** Quantification of YFP induction as a function of light stimulus duration for the *fos-tubulin* SynIEG (in **c**) and the *fos*-Fra1deg-*fos* SynIEG implementing Fos-specific mRNA- and protein-level regulation (in **d**). The fold-change in YFP fluorescence was quantified from NIH 3T3s that also expressed the iLID-OptoSOS system and were stimulated with either transient (20 min) or sustained (90 min) blue light. All curves indicate the mean ± SEM for *n* = 10 cells (panel **c**, sustained), *n* = 6 cells (panel **c**, transient), *n* = 22 cells (panel **d**, sustained), and *n* = 23 cells (panel **d**, transient). **e** Schematic of combinatorial control over *BTG2* induction: Erk stimulation induces btg2 transcription but not protein accumulation, whereas DNA damage induces both. The mechanism underlying this difference is unknown. **f** Representative images of YFP levels in NIH3T3 clonal lines expressing various SynIEGs and stimulated with 10% serum. Images show YFP levels just before and 3 h after serum stimulation and scale bar is for 10 μm. **g** Quantification of the area under the curve (AUC) of YFP induction after serum stimulation for the clonal SynIEG cell lines shown in **f**, as well as for *fos-tubulin* as an additional control (see Methods for quantification details). Each point represents the average of 20–30 cells from an independent experiment. **h** Quantification of YFP fluorescence in NIH3T3 cells harboring the *fos*-*btg2* SynIEG and stimulated with 10% serum after being transduced with nothing (*n* = 20 cells), miR-21 (*n* = 17 cells), or the miR-21 sponge (*n* = 21 cells). Single-cell traces are shown in lighter color, with the mean response shown as a bold line. Additional clones tested for miR-21 effect are shown in Supplementary Fig. 8.

We next tested whether SynIEGs could be used to define the regulatory steps that enable target genes to selectively respond to dynamics of upstream signaling. We focused on the *FOS* gene as its regulation is a canonical example of dynamic decoding: sustained, but not transient, activation of the Ras/MAPK pathway triggers an increase in Fos protein levels^11,12^. We thus set out to construct variants of *FOS* SynIEGs to recapitulate its dynamic filtering (Fig. 3a).

Dynamic filtering by *FOS* is thought to depend on gene expression as well as two forms of post-transcriptional regulation, Erk-dependent destabilization of the *FOS* 3′ UTR^26^ and stabilization of Fos protein^11^, although additional forms of regulation have not been ruled out. We thus tested whether a minimal set of regulatory elements would indeed be capable of dynamic filtering. We combined the *FOS* promoter, *FOS* 3′UTR, and a well-characterized Erk-responsive degron from the FOS family member Fra1^27^ to build a SynIEG termed *fos-*Fra1^deg^*-fos* (Fig. 3b). The *fos-tubulin* SynIEG was used as a control lacking both forms of post-transcriptional regulation. We introduced both SynIEGs into “chassis” NIH3T3 cells that were also sorted to express an optogenetic system to turn on the Erk pathway, the blue light-sensitive OptoSOS system (iLID-OptoSOS)^16,17^, which enables us to precisely control the dynamics of pathway activity by varying the duration of illumination (Supplementary Fig. 5a). In the absence of blue light, SSPB-SOScat is cytosolic and inactive, whereas blue light stimulation induces SSPB-SOScat membrane localization (Supplementary Fig. 5b**)** and activates Ras/Erk signaling (Supplementary Fig. 5c–e).

We incubated OptoSOS-SynIEG cells in serum-free media for 6 h, applied either a 20-min pulse of light (“transient”) or continuous illumination (“sustained”), and monitored YFP induction over time (Fig. 3c, d). For *fos-tubulin* cells, both light stimuli triggered similar increases in YFP fluorescence over time (Fig. 3c), likely because both sustained and transient Erk-activating stimuli cause an identical pulse of transcription, as observed earlier (Fig. 2, Supplementary Fig. 4) and in prior studies^21^. However, we found that in the *fos*-Fra1^deg^-*fos* clonal cell line, sustained light drove higher protein accumulation than a 20-min light pulse (Fig. 3d). It thus appears that the two post-transcriptional regulatory connections contained in the *fos*-Fra1^deg^-*fos* SynIEG (Erk-dependent protein stabilization and mRNA degradation) are indeed sufficient to confer dynamic selectivity for sustained Erk stimuli.

**Fig. 4:**
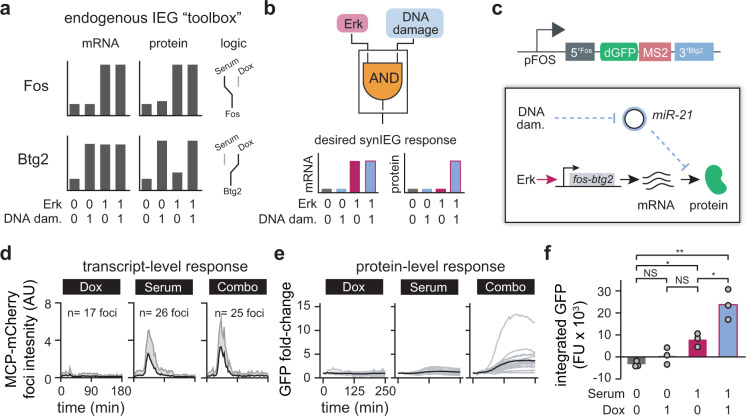
A SynIEG-based AND gate triggers target gene expression in response to Erk and DNA damage. **a** Cartoon schematic of prior knowledge of *FOS* and *BTG2* regulation in response to growth factor stimulation (serum) and DNA damage (doxorubicin; Dox). Fos transcription and protein accumulation are both triggered by serum stimulation irrespective of DNA damage (upper panels). In contrast, btg2 transcription is induced by both DNA damage and serum stimulation, yet only DNA damage triggers Btg2 protein accumulation. **b** Schematic of a synthetic AND gate responding to both serum stimulation and DNA damage. By combining a *FOS*-like transcriptional response with a *BTG2*-like protein response, target gene induction would be triggered only when both stimuli are supplied. **c** Experimental implementation of the AND-gate SynIEG. Transcription of the *fos-btg2* SynIEG is induced only by serum stimulation, not DNA damage, due to its use of the *FOS* promoter. However, DNA damage is required for protein accumulation through the use of the *BTG2* 3′UTR. **d** Transcriptional response of the *fos-btg2* SynIEG. The brightness of individual nuclear MCP-mCherry foci was quantified (mean + SD) in cells treated with doxorubicin (*n* = 17 foci, 17 cells), serum (*n* = 26 foci, 12 cells), or their combination (*n* = 25 foci, 10 cells). **e** GFP induction from the *fos-btg2* SynIEG. Nuclear dGFP levels were quantified from cells stimulated with doxorubicin, serum, or their combination. Single-cell traces are shown in gray, with the mean response shown as a black line. **f** Quantification of the area under the curve (AUC) of dGFP induction for the clonal SynIEG cell lines shown in **d** and **e** after stimulation with serum, doxorubicin, or their combination (see “Methods” for quantification details). Each point represents the mean of at least 20 cells in a single experiment, with each bar representing the mean of three independent replicate experiments. Statistics are derived using an unpaired two-sided *t*-test (*p < 0.05, **p < 0.01, ***p < 0.001).

We also observed that the fold-change in SynIEG protein expression was relatively low: 40% in the case of *fos-tubulin* and 20% in the case of *fos*-Fra1^deg^-*fos* cells over a 3 h timecourse (Fig. 3c, d). We conjectured that this low fold-change may reflect the high stability of the YFP transgene, leading to its failure to “reset” to a low level in starvation media prior to serum addition. To further explore this hypothesis, we compared *fos-fos* SynIEG cells to a previously derived NIH3T3 clone with a YFP-tagged endogenous *FOS* gene^21^. Both cell lines were starved in serum-free media for 5 h and then serum-stimulated to trigger YFP expression. Indeed, although the fold-change increase in endogenous *FOS* levels was higher than the *fos-fos* SynIEG (Supplementary Fig. 6a), quantifying the integrated raw fluorescence revealed that the *fos-fos* SynIEG had comparable, if not higher, amounts of total protein induction (Supplementary Fig. 6b**)**. These results informed design of subsequent SynIEG circuits using destabilized fluorescent reporters, described further in Fig. 4.

### SynIEGs reveal the basis of *BTG2*’s stimulus-dependence

We next turned our attention to the stimulus-specific gating of *BTG2*, where the requirements for protein expression in response to various upstream signals are still poorly understood^28^. *BTG2* transcription is induced by many cellular stimuli, from growth factors^21^ to DNA damage^29,30^ and other stresses^31^. Different cellular stimuli also appear to regulate *BTG2* at transcriptional and post-transcriptional steps. For example, we previously observed that Btg2 protein levels were unchanged in NIH3T3 cells after growth factor stimulation despite sustained induction of btg2 mRNA. In contrast, DNA damage produced by the topoisomerase inhibitor doxorubicin triggered both transcription and protein-level induction of Btg2^21^ (Fig. 3e). We reasoned that by constructing SynIEGs incorporating various elements of the *BTG2* gene, we might be able to identify the source of stimulus-specific gating and generate a modular component that could be repurposed for designing additional logic functions.

Our first goal was to determine whether this block to Btg2 protein accumulation after growth factor signaling occurred at the mRNA level (e.g. by regulating mRNA degradation or blocking translation) or at the protein level (e.g. by regulating protein degradation). To test for mRNA-level regulation, we constructed a series of SynIEG variants that lacked any Btg2 protein sequence but harbored various combinations of *BTG2* and *FOS* UTR sequences (termed the *btg2-btg2*, *btg2*-*fos, fos-btg2*, and *fos-fos* SynIEGs). We derived clonal cell lines for each SynIEG, starved each for 4–6 h, and then stimulated with serum to monitor YFP induction, using identically treated *fos-tubulin* SynIEG cells as controls (Supplementary Fig. 7). We found that only the SynIEGs containing the *BTG2* 3′ UTR failed to accumulate YFP in response to serum stimulation (Fig. 3f, g), whereas all other SynIEGs exhibited similar levels of YFP accumulation. The *BTG2* 3′ UTR could in principle limit YFP accumulation either by triggering mRNA degradation or blocking protein translation. However, we previously found that endogenous btg2 mRNA levels remain high for hours after serum stimulation even though no protein is made, whereas DNA damage triggers both mRNA- and protein-level induction^21^. Together, these data demonstrate that RNA-level regulation is sufficient to explain Btg2’s paradoxical response of high serum-induced expression with no protein accumulation and suggests a mechanism of translational repression mediated by the *BTG2* 3′ UTR.

Prior reports indicate that over a dozen microRNAs may bind to the *BTG2* 3′ UTR and regulate Btg2 protein expression in various cell types and cancers^32–36^. We focused on miR-21 as a candidate regulator because it is upregulated after growth factor stimulation (in contrast to the majority of microRNAs)^37^, and because miR-21 has been implicated in Btg2 regulation in other contexts^34,35^. To test whether miR-21 is responsible for Btg2’s translational repression after acute growth factor stimulation, we generated cell lines that overexpress miR-21 or an antisense “sponge” to titrate away endogenous miR-21, reasoning that these constructs should have opposite effects on YFP accumulation. We created lentiviral expression vectors containing the U6 promoter driving either miR-21 or the antisense sponge, followed by a constitutive CMV promoter driving the expression of TagBFP-NLS to label cells that were successfully transduced. We found that SynIEG cells transduced with the miR-21 sponge exhibited YFP induction after serum stimulation, whereas mock-transduced or miR-21-transduced cells failed to accumulate YFP accumulation (Fig. 3h, Supplementary Fig. 8). These data confirm a model whereby growth factor signaling drives *BTG2* transcription but miR-21 blocks Btg2 translation from this mRNA. Overall, our results are consistent with prior reports of *BTG2* repression in other cellular contexts^35^, and with reports indicating that miR-21 can block translation of target mRNAs^38–41^. More broadly, we find that SynIEGs provide a flexible system for studying the decoding of cell signaling stimuli at multiple steps along the central dogma, from transcriptional induction (e.g., transcriptional kinetics via MS2/MCP imaging) to translational regulation (e.g., testing different 5′ and 3′ regulatory sequences) to protein-level regulation (e.g., Erk-dependent stabilization of the Fra1 degron).

### Engineering a SynIEG sensor of mitogens and DNA damage

In addition to dissecting the regulation of natural immediate-early genes, SynIEGs are also well suited for engineering signaling-responsive circuits with previously unreported, desired response functions. To explore this possibility, we next set out to develop a SynIEG which implements a signaling-response function that has not been previously described for any immediate-early gene: an AND gate in which growth factor stimulation and a second stimulus, DNA damage, are both co-required to induce gene expression.

Our strategy for engineering an AND-gate SynIEG relies on repurposing and combining the *FOS* and *BTG2* regulatory elements characterized in the preceding sections. We previously observed that the *FOS* gene is transcribed in response to growth factor stimulation, regardless of whether DNA damage is present^21^ (Fig. 4a). In contrast, 3′ UTR-based translational repression of the *BTG2* gene is abolished in cells exposed to DNA damage delivered either alone or in combination with serum^21^ (Fig. 4a). When combined, these two regulatory elements could thus result in a circuit that requires two inputs: serum to promote transcription and DNA damage to relieve translational repression (Fig. 4b). All of the components required for implementing such an AND gate would be encoded in a single transgene, underscoring the efficiency of our approach.

We constructed a candidate AND-gate SynIEG by combining the *FOS* promoter and 5′ UTR, the *BTG2* 3′ UTR, and a coding sequence driving expression of a destabilized GFP (dGFP) variant. We chose dGFP over our prior YFP reporter to “reset” the circuit to a lower baseline during incubation in serum-free media prior to stimulation, potentially increasing the fold-change in transgene expression (Fig. 4c). A constitutive CMV promoter driving TagBFP expression was placed downstream in the same integration vector to enable circuit-independent selection of PiggyBAC-transduced NIH3T3 cells. We then performed single-cell sorting to derive two independent clonal cell lines expressing the candidate AND-gate SynIEG.

We characterized mRNA- and protein-level responses from cells expressing the candidate AND-gate SynIEG in response to three classes of stimuli: doxorubin (DNA damage alone), 1% serum (growth factor alone), or doxorubicin + 1% serum (DNA damage AND growth factor). At the transcriptional level, we observed pronounced MCP foci in AND-gate SynIEG cells treated with serum regardless of whether doxorubicin was present (Fig. 4d), a result that was consistent with our prior observations from the *FOS* endogenous locus^21^. At the protein level, dGFP failed to accumulate or responded weakly to either doxorubicin or serum alone, whereas the combination of serum and doxorubicin triggered strong dGFP accumulation (Fig. 4e, Supplementary Fig. 9a). We further verified that the overall change in dGFP fluorescence (area under the curve; AUC) was substantially increased by the combination of serum and doxorubicin compared to either input alone in both independently derived clonal cell lines (Fig. 4f, Supplementary Fig. 9b). Additionally, a control SynIEG (*fos*-*fos*) did not exhibit AND-gate logic, ruling out the possibility that the AND-gate-like logic resulted from factors that were extrinsic to our circuit (Supplementary Fig. 9c). Taken together, these data demonstrate the successful construction of a gene that synergistically responds to the combination of mitogen and DNA damage inputs using immediate-early gene components. Expression of a desired genetic payload is enabled when growth factor stimulation induces SynIEG transcription and DNA damage relieves microRNA-mediated translational repression. Despite this success, we note that weak activation in response to serum alone prevents its characterization as a perfect AND gate. Further improvements might suppress this weak activation, such as incorporating additional miR-21 binding sites in the 3′ UTR to enhance translational repression. Nevertheless, because super-additive logic has not to our knowledge been reported for any endogenous immediate-early gene, our results demonstrate that the SynIEG platform can be used to engineer new signal-response relationships as well as to dissect endogenous IEG regulatory links.

## Discussion

There is still a critical gap in our understanding of mammalian signal decoding; understanding how target genes implement complex signal processing functions by combining specific molecular interactions^4^. To address this gap, we set out to build mammalian gene cassettes that allow one to simultaneously monitor the transcription and translation of a gene while modulating its various components (e.g., protein degrons, UTRs, enhancer/promoter sequences, etc.) (Fig. 2a). After optimizing gene delivery, we quantitatively characterized SynIEG transcription to ensure that their transcriptional regulation was similar to endogenous IEGs (Fig. 2c, d, Supplementary Fig. 4, Supplementary Movie 1). We then used SynIEGs to recapitulate the response of the *FOS* gene to dynamic Ras/Erk stimuli (Fig. 3a–d) and to dissect how *BTG2* responds selectively to DNA damage but not growth factor stimulation despite similar transcriptional behavior in each case (Fig. 3e–h). In both cases, our SynIEG studies revealed regulatory links between signaling pathways and downstream target gene expression that act on multiple nodes of the central dogma (e.g., induction of *BTG2* transcription but translational inhibition caused by the *BTG2* 3′ UTR). It should be noted that these results absolutely required the ability to monitor cellular responses at both transcriptional and post-transcriptional steps, a capability that is frequently lacking in standard reporter gene assays. Future studies using SynIEGs can enable the characterization of the signaling pathway to gene expression interface and will shed light on how cells interpret complex biochemical cues to induce a variety of cell fates and how this signal decoding may change as a function of disease.

Based on these results, we conjectured that immediate-early genes might also serve as a useful engineering substrate for constructing mammalian cell signaling decoders with desired stimulus–response relationships. Indeed, we found that by combining elements from known immediate-early genes—the *FOS* serum-responsive promoter and the *BTG2* DNA damage-responsive 3′UTR—it was possible to construct a synergistic response that triggers a fourfold increase in protein levels only in response to combined serum stimulation and DNA damage (Fig. 4). A striking feature of this target gene is its simple construction from two elements, a promoter and 3′ UTR from two separate endogenous IEGs, without additional fine-tuning or optimization. This simplicity follows from the fact that both elements are regulated at distinct steps of the central dogma. SynIEG protein accumulation occurs only if transcription is activated (via growth factor stimulation) and translation is de-repressed (via DNA damage and microRNA regulation). In contrast, engineering AND-gate logic at a single step (e.g. engineering two transcription factors to be mutually required for transcription) often requires complex engineering of three-body interactions (two protein domains with one another and a DNA sequence)^42^. In addition to constructing logic gates, multi-step regulation is likely to be essential for selectively responding to specific signaling dynamics. For instance, feed-forward loops acting on different timescales have been shown to play crucial roles in discriminating sustained from transient stimuli^9,43^.

We propose that SynIEGs implementing previously unreported decoding functions should find utility as reporters of complex endogenous signaling conditions (e.g. marking cells in which some Pathway 1 and Pathway 2 are both active), or as circuits to control the function of engineered cells^44^. It is known that immediate-early genes are expressed broadly in many tissues, and have extremely potent and fast signaling-induced responses, making them likely to be expressed highly in many contexts^45,46^. Furthermore, our work has revealed that SynIEGs display homogeneous transcriptional responses after growth factor stimulation, even after random insertion throughout the genome using the PiggyBAC transposase (Fig. 2c, d, Supplementary Figs. 3 and 4). This corroborates recent work in which local chromatin structure was seen not to affect transcription of the actin gene^47^, and where gene expression was largely insensitive to some large-scale genome rearrangements^48^. All of this suggests that SynIEGs might indeed serve as a predictable platform for mammalian signaling-induced response regulation, but future studies will need to look carefully at other classes of genes before such engineering efforts are applied more broadly. Although SynIEGs as they stand may not be perfect tools as they have modest fold-changes because they are currently limited by the endogenous IEG promoter strengths (Supplementary Fig. 6), improvements can simply be made by amplifying protein output by using strong transcriptional activators (i.e. Gal4, Qf, SynTF, etc.)^49,50^. As the field of synthetic biology gets closer to medical applications, especially in the burgeoning field of immunotherapy, cells will need to be engineered to interpret increasingly complex extracellular information^44^. In the future, SynIEGs implementing multi-step regulatory relationships could provide a valuable tool for achieving this goal.

## Methods

### Plasmid construction

We cloned all of our constructs/synthetic gene circuits into previously published pHR lentiviral expression plasmid^2^ or into Piggybac plasmid^51^. For large PCR products (>7500 bp), GXL polymerase was used, followed by overnight DPN1 digestion while for smaller PCR products, HiFi polymerase from Clontech was utilized. Sequences for pFOS, *FOS* 5′ UTR, *FOS* 3′ UTR, *BTG2* 5′ UTR, *BTG2* 3′ UTR, and *TUBULIN* 3′ UTR (can be found in Supplementary Note 1) were obtained via PCR from genomic DNA obtained from NIH3T3 cells made using Epicentre’s QuickExtract. Sequences for BFP, YFP, MS2 loops, and OptoSOS were obtained from plasmids published previously^21,52,53^. Destabilized GFP (dGFP) was obtained as a generous gift from the Reya lab (Addgene: 14715). Fra1 degron was based of FIRE reporter^27^, which was encoded within an IDT gBlock (sequence can be found in Supplementary Note 1). PCR products were then run on an agarose gel, purified using the Takara Bio’s Nucleospin gel purification kit. Final plasmids were constructed using Takara Bio’s Infusion reagent, amplified in Stellar chemically competent *E. coli*, and DNA was extracted using Qiagen miniprep kits.

Construction of a microRNA expression plasmid was made based off of lentiCRISPR v2 plasmid^54^ in which the U6 promoter is followed by *Age*I and *Eco*RI restriction enzyme cut sites which results in a 7125 and 1875 bp band on a agarose gel. The larger piece was excised and purified. miR-21 sequence (*agttgtagtcagactattcgat*) and miR-21 inhibitor sequence (*tcaacatcagtctgataagcta*) were encoded in duplexed primers with CCGG 5′ overhang for the top strand and AATT 5′ overhang for the bottom strand. T4 ligase was then used to ligate the microRNA sequences into backbone of the plasmid. The resulting plasmid was transformed into Stellar chemically competent *E. coli* and DNA was extracted using Qiagen miniprep kit.

All plasmid verification was done by restriction enzyme digestion and Sanger sequencing by submitting through Genewiz. All plasmids used in this study can be found in Supplementary Table 1.

### PiggyBac integration

NIH 3T3s to be integrated were plated 24 h prior to transfection. In all, 2.08 μg of the plasmid to be integrated along with 0.41 μg of the piggybac helper plasmid were co-transfected into the cells using Liptofectamine LTX with Plus reagent. Cells were selected using FACS for YFP expression after waiting at least 3 days post-transfection. SynIEGs from Figs. 2 and 3 were integrated into the “Chassis” clonal line described in ref. ^21^. Briefly, the Chassis clonal line is a NIH3T3 cell line with BFP-Erk, MCP-mCherry, and H2B-iRFP. The *fos*-dGFP*-btg2* SynIEG was integrated into wild-type NIH 3T3s. Cells then underwent single-cell sorting to isolate clonal cell lines bearing the various SynIEG variants.

### Lentivirus production

HEK 293Ts were plated in a six-well plate at ~40% confluency at least 12 h before transfection. The cells were then co-transfected with 1.5 μg of the pHR vector of interest along with 1.33 and 0.17 μg of CMV and pMD packaging plasmids, respectively, using Fugene HD (Promega). Virus was collected after 48 h post-transfection and filtered through a 0.45-mm filter. To the ~2 mL of viral media, 2 μL of polybrene and 40 μL of 1 M HEPES were added. Cells to be infected were plated at 40% confluency in a six-well plate at least 12 h before infection and then 200–500 μL of viral media was added to the cells. Twenty-four hours post transduction, virus containing media was replaced with fresh media. Cells were then incubated for at least another 24 h before sorting using FACS Aria or being placed on the microscope for experiments.

### Cell line maintenance and preparation for imaging

NIH 3T3s were grown in Dulbecco’s modified Eagle’s medium (DMEM) plus 10% fetal bovine serum (FBS) in Thermo Fisher Nunc Cell Culture Tissue Flasks with filter caps at 37 °C and 5% CO_2_. Cells to be imaged were plated into InVitro Scientific’s 96-well, black-walled, 0.17-mm high-performance glass bottom plates. In all, 10 μg/mL of fibronectin diluted in phosphate-buffered saline (PBS) was placed on the wells, washed off, and then cells were plated in DMEM with 10% FBS at least 12 h prior to imaging. Between 4 and 6 h prior to imaging, cells were placed in serum-free media (DMEM with 0.00476 mg/mL HEPES). Fifty microliters of mineral oil was pipetted onto the wells right before placing onto the scope to prevent media from evaporating.

### Imaging and optogenetic stimulation hardware

Cells were maintained at 37 °C with 5% CO_2_ for the duration of an imaging experiment. Confocal microscopy was performed on a Nikon Eclipse Ti microscope with a prior linear motorized stage, a Yokogawa CSU-X1 spinning disk, an Agilent laser line module containing 405, 488, 561, and 650 nm lasers, ×60 oil emersion objective, and an iXon DU897 EMCCD camera.

For optogenetic microscope experiments, blue light from the XLED1 system was delivered through a Polygon400 digital micromirror device (DMD; Mightex Systems) to control the temporal dynamics of light inputs. We applied specific temporal patterns to an image by drawing ROIs within the Nikon Elements software package and using custom macros to turn on and off the light. To attenuate 450 nm light, we dithered the DMD mirrors to apply light 50% of the time, and set our 450 nm LED to 50% of its maximum intensity.

### Drug treatments

Drug additions were done with a 200 μL gel loading pipette directly onto cells while they were on the microscope. Drugs were pre-diluted to a 1:10 stock concentration additions. Final concentrations for drugs were cycloheximide (100 μg/mL), doxorubicin (860 nM), FBS (10% by volume for all experiments except for the AND-gate experiments which was done at 1% by volume).

### Transcriptional burst analysis

Bursting MCP foci were imaged and quantified using a protocol adapted from ref. ^21^. Briefly, seven z-stack slices spanning 4.5 μm (0.8 μm between z-slices) which was centered on the middle of the nucleus. This z-stack was max projected to allow all of the bursts to be visualized on a single plane. Positional information was tracked using the measure tool in Fiji. MATLAB code was used to take in the positional information, fit a two-dimensional Gaussian to the identified region, and finally calculated the integrated area under the fitted Gaussian as the burst intensity. This code can be found in supplementary MATLAB file and a schematic of this pipeline can be found in Supplementary Fig. 2.

### Microscopy data analysis

ND2 files from Nikon Elements software were imported into ImageJ. The measure tool was used to quantify mean intensity of the nuclei of cells of interest. These files were saved and then imported into R to do statistical analysis and graphing. Area under the curve analysis was done by first averaging the first two timepoints to set the baseline. The area was then calculated by using the following formula:

$${\rm{AUC}} = \mathop {\sum}\limits_{t = 0}^{t = t_{{\rm{final}}}} {({\rm{intensity}}\left( t \right) - {\rm{initial}})}.$$

The code for this analysis can be found in Supplementary code.

### Statistics and reproducibility

Data in figure legends include whether data are represented as mean ± SEM, mean + SD, or mean ± SD as well as sample size. For AND gate experiments in Fig. 4 and Supplementary Fig. 9, a two-sided unpaired *t*-test was applied between starve and each of the separate conditions as well as between serum and doxorubicin alone and the combination of serum and doxorubicin. For miR-21 statistical testing in Supplementary Fig. 8b, a paired, two-tailed *t*-test was applied as we wanted to compare within clones for both positive and negative effects on YFP protein accumulation in response to the different miR-21 statuses. A two-sided Student’s *t*-test was deemed appropriate because we sought to test for both positive and negative effects on YFP protein accumulation in response to the different stimuli.

### Reporting summary

Further information on research design is available in the Nature Research Reporting Summary linked to this article.

## Supplementary information

Supplementary Information

Description of Additional Supplementary Files

Supplementary Data 1

Supplementary Data 2

Supplementary Data 3

Supplementary Movie 1

Reporting Summary

## Data Availability

Raw data for all experiments have been included as Supplementary Data 1. MATLAB and R scripts used for data analysis are shown in Supplementary Data 2 and 3, respectively. Additional data for experiments are available upon reasonable request.

## Code availability

The code for burst analysis and a test dataset is supplied as Supplementary Data 2 and a walkthrough of what the code achieves is represented in Supplementary Fig. 2. The code for analyzing protein accumulation experiments and a test dataset is supplied as Supplementary Data 3.
