# Supplementary material for: Engineering combinatorial and dynamic decoders using synthetic immediate-early genes
Source: Commun Biol. 2020 Aug 13;3:436. doi: 10.1038/s42003-020-01171-1 (PMC7426417; doi:10.1038/s42003-020-01171-1)
Supplement: Supplementary file 1 — Supplementary Information [file 42003_2020_1171_MOESM1_ESM.pdf]

Supplementary Materials for

Engineering combinatorial and dynamic decoders using synthetic immediate-early genes

Pavithran T. Ravindran[1,2], Maxwell Z. Wilson[2,3], Siddhartha G. Jena[2], Jared E. Toettcher[2]

This PDF file includes:

Supplementary Figures 1-9
Supplementary Table 1
Supplementary Note 1
Supplementary References

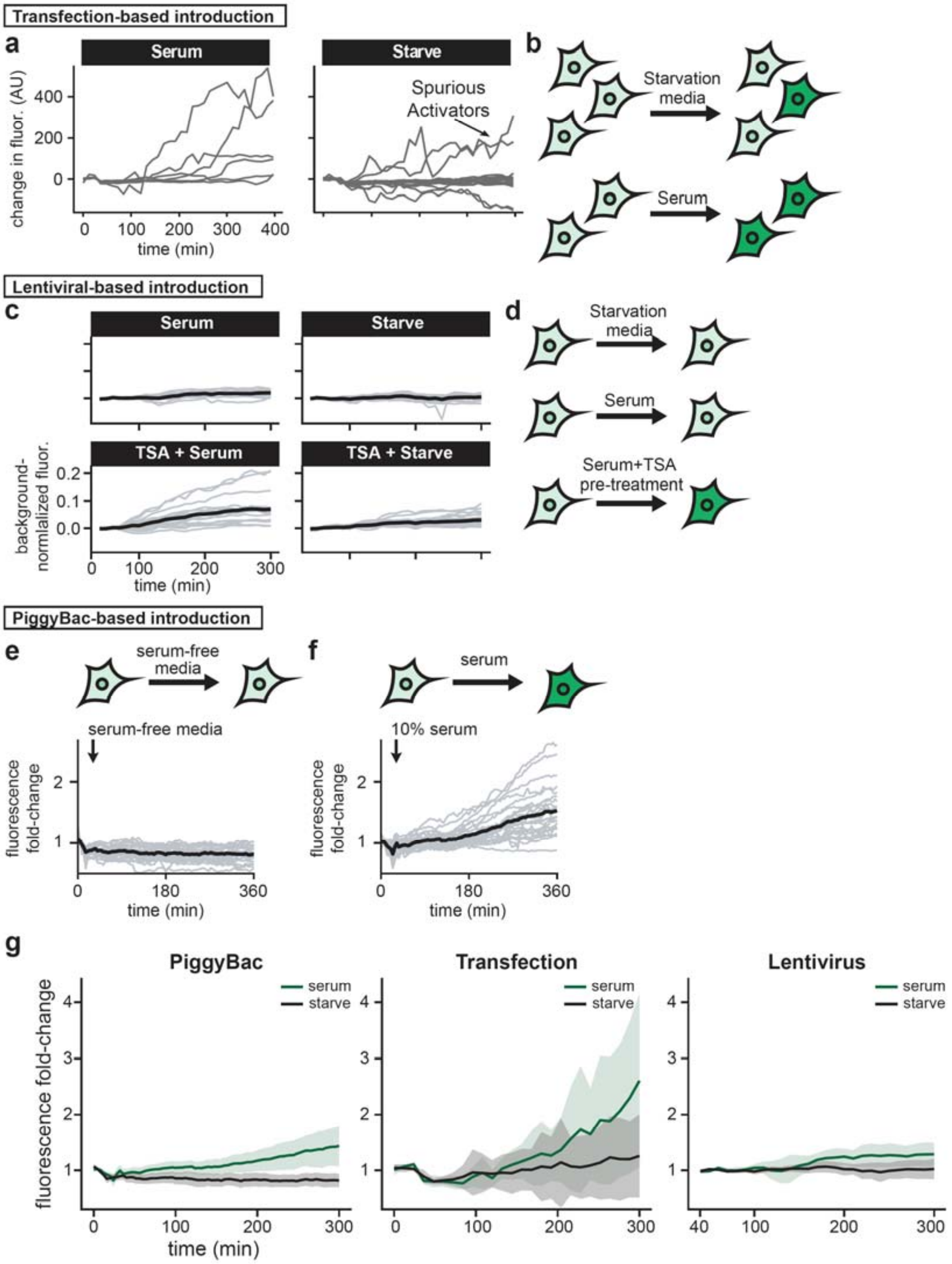

Supplementary Figure 1. Comparison of methods to introduce synthetic target genes into target cells. (**A**) Quantification of YFP fluorescence in NIH 3T3 after being transfected with *fos-tub* SynIEG and treated with either serum (n=6 cells) or growth factor free media (n=20 cells). Arrow denotes some of the traces in the starvation case in which YFP intensity increases. (**B**) Model of transfection based method of delivery for SynIEGs. Also serum induced cells increase in YFP fluorescence, there is spurious activation of SynIEGs in the serum free case. (**C**) Dynamic traces of NIH 3T3s that were lentivirally integrated with *fos-tubulin* SynIEG. Means

(dark black line) and single cell traces (light gray lines) are shown for cells that were either given starvation media (n=16 cells), serum stimulation (n=10 cells), pre-treated with TSA and then given starvation media (n=20 cells) or pre-treated with TSA and then treated with serum (n=19 cells). (**D**) Model for lentiviral transduction of SynIEGs: after silencing occurs, neither starvation media nor serum induces YFP accumulation but with the addition of trichostatin A, YFP is now inducible. (**E-F**) Dynamic single cell traces (light gray lines) with mean shown in dark black line for NIH 3T3s containing the *fos-tubulin* synIEG after PiggyBac integration for cells that received starvation media (in **E**; n=27 cells) and serum (in **F**; n=26 cells). (**G)** Direct comparison of all three methods. Data from (A), (C) and (E-F) are presented with foldchange over initial YFP expression over time and standard deviation shown in the shading around each line.

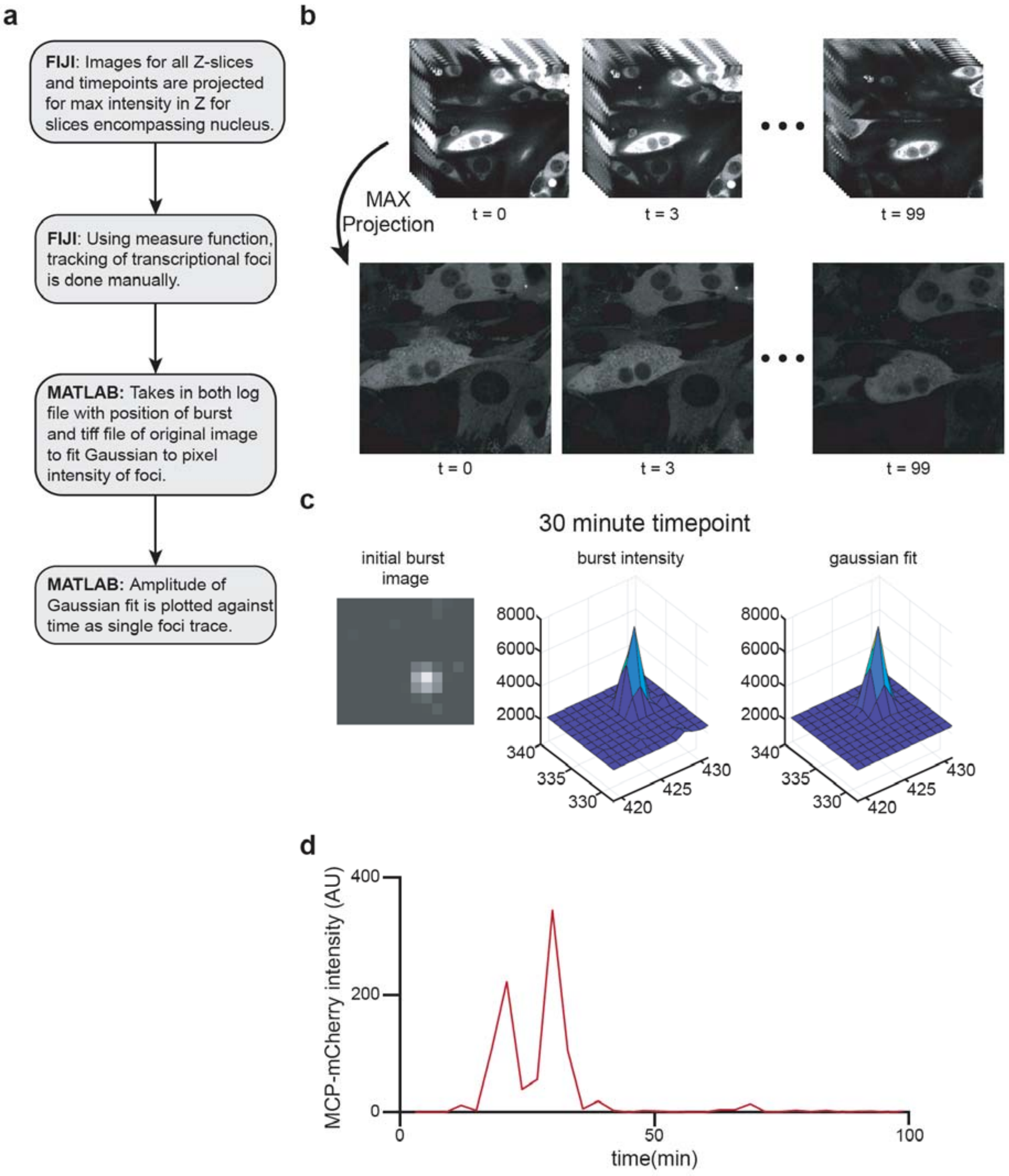

Supplementary Figure 2. Data processing pipeline for microscopy data of transcriptional foci. **(A)** Flow-through diagram of the process of going from z-stack tif files to foci traces using combination of FIJI and MATLAB. **(B)** Examples z-stack tif file for data from **Fig 2C-D** before and after maximum projection in z**. (C)** Example of Gaussian fit for foci intensity. **(D)** Example trace of foci intensity over time.

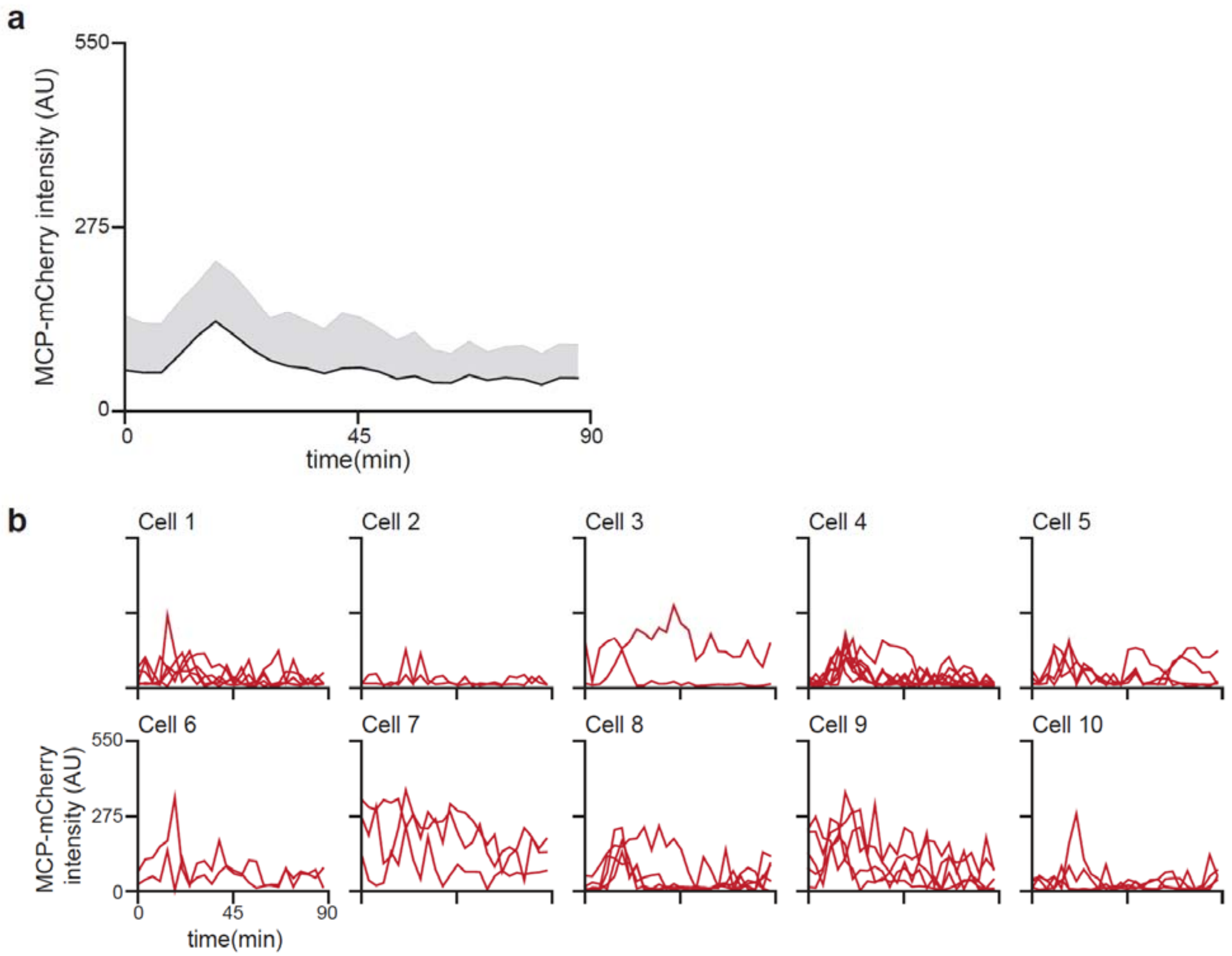

Supplementary Figure 3. Inter- and intracellular variation in transcriptional foci. (A) Transcriptional response of the *fos-btg2* SynIEG. The brightness of individual nuclear MCP-mCherry foci was quantified (mean + S.D.) in cells treated with serum (n= 40 foci, 10 cells). **(B)** Transcriptional response of the *fos-btg2* SynIEG for cells shown in **(A)** for each individual foci broken up by corresponding cell.

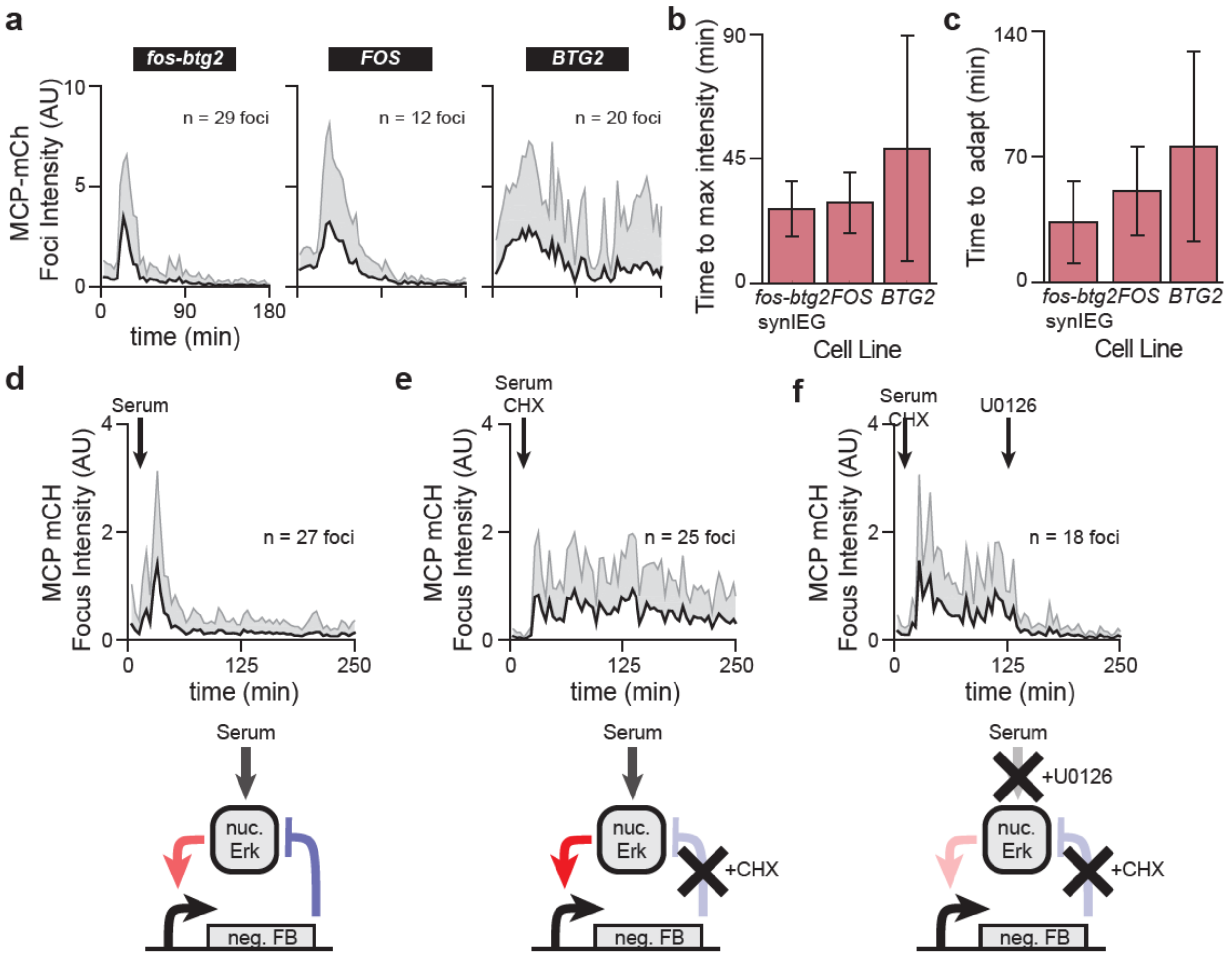

Supplementary Figure 4. synIEGs recapitulate the dynamics and regulation of endogenous IEGs. (**A**) Mean + S.D for transcriptional foci intensity after serum stimulation for NIH 3T3s either containing YFP-MS2 tag on endogenous *BTG2* (n = 20 foci, 14 cells) or *FOS* (n= 12 foci, 11 cells) endogenous gene or *fos-btg2* SynIEG (n = 29 foci, 10 cells). (**B**) Bar graph showing mean± S.D for the time until maximum intensity of transcriptional foci after serum stimulation for cells quantified in **A**. (**C**) Bar graph showing mean± S.D for the time until adaptation (defined as the time after reaching maximum intensity when the burst intensity is 50% of the maximum) for cells quantified in **A**. (**D-E**) Quantification of MCP-mCherry nuclear foci (mean + S.D.) in *fos-btg2* SynIEG cells treated with serum (in **D**; n= 27 foci, 12 cells), serum + cycloheximide (in **E**; n=25 foci, 11 cells) or serum and cycloheximide followed by addition of the MEK inhibitor U0126 after 90 min (in **F**; n= 18 foci, 6 cells). Underneath each graph is a schematic of the interactions occurring between nuclear Erk, and production of its negative feedback.

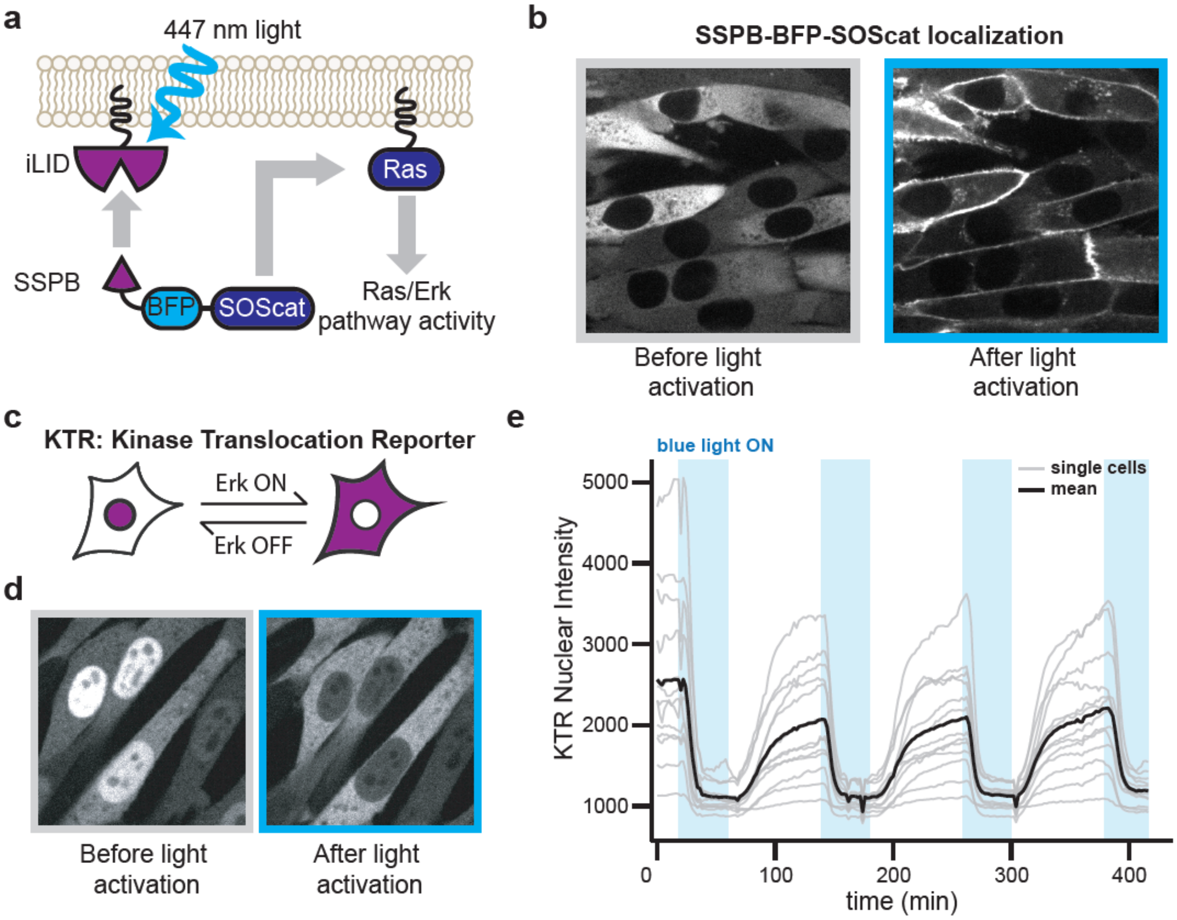

Supplementary Figure 5. The iLID-OptoSOS system allows for transient and sustained activation of MAPK activity in synIEG-expressing cell lines. (**A**) Diagram of the OptoSOS system where blue light dimerizes iLID and SSPB, recruiting SOScat to the membrane, activating the Ras-Erk pathway. (**B**) Representative image of BFP-SOScat before and after blue light stimulation. (**C**) Schematic of the kinase translocation reporter (KTR) that is present in the nucleus when Erk is off and in the cytoplasm after Erk is on. (**D**) Representative images of the iRFP channel for NIH 3T3s expressing the OptoSOS – iLID/SSPB system as well as KTR fused to iRFP. Before light, cells have Erk off as evidenced by nuclear KTR and after blue light stimulation, cells have turned Erk on. (**E**) Dynamic traces of 13 NIH 3T3 cells expressing the OptoSOS – iLID/SSPB system as well as KTR fused to iRFP where dark black line represents the mean and gray traces are individual cells. Blue bars show where the blue DMD was turned on. Cells were given 10 minutes no light, 20 minutes blue light and then 30 minute no light cycles over the course of the experiment.

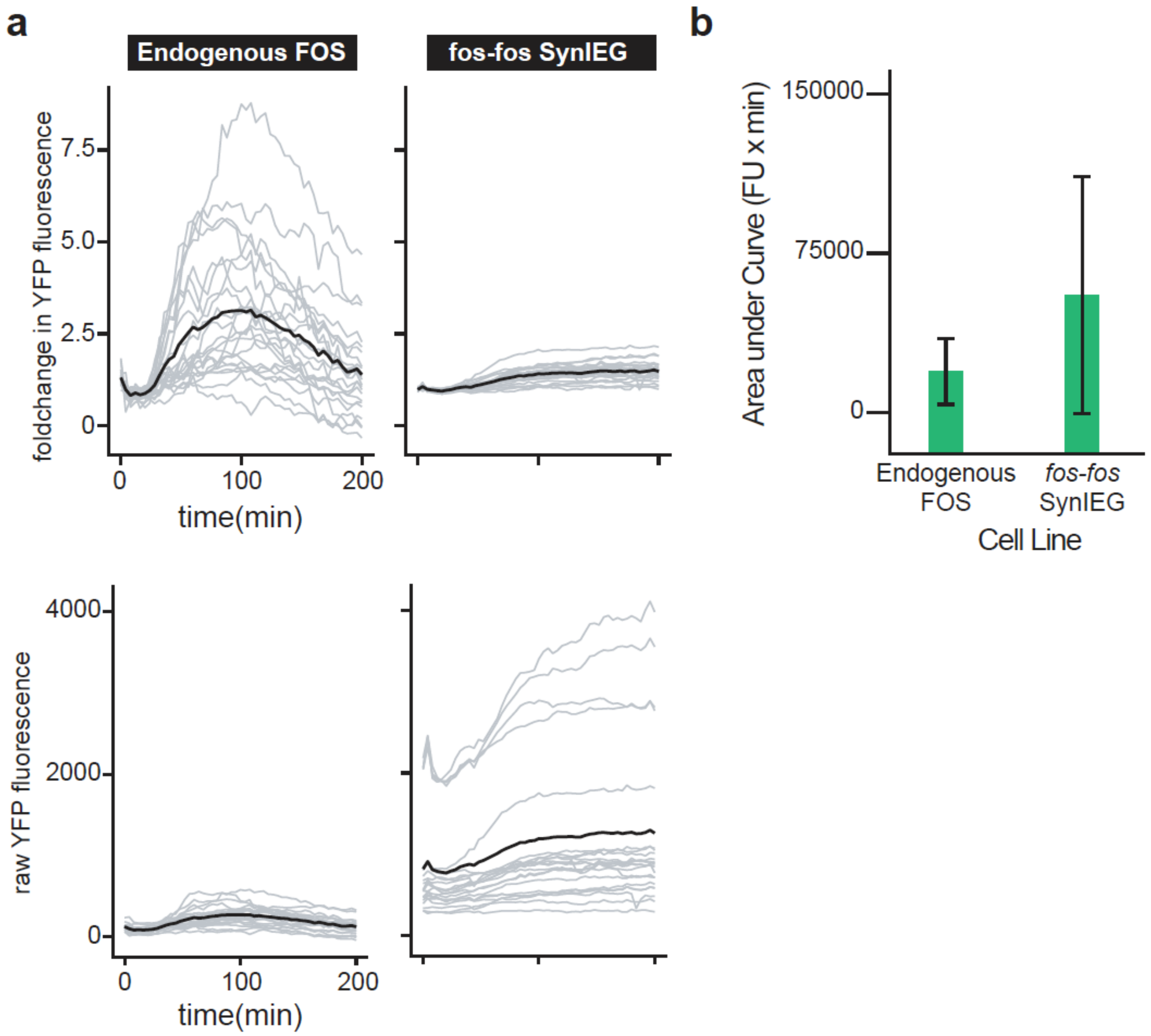

Supplementary Figure 6. Comparison of protein expression between endogenous *FOS* and *fos-fos* SynIEG. (A) YFP fold-change traces for cells with endogenous *FOS* tagged with MS2-YFP (n=23 cells) and *fos-fos* synIEG (n=22 cells). Cells were treated with 10% serum after being in growth-free media for 5 hours. Gray traces are single cells and dark black line is the mean. **(B)** Quantification of the area-under-the-curve (AUC) of YFP induction after serum stimulation for the cells shown in **A** (see Methods for quantification details).

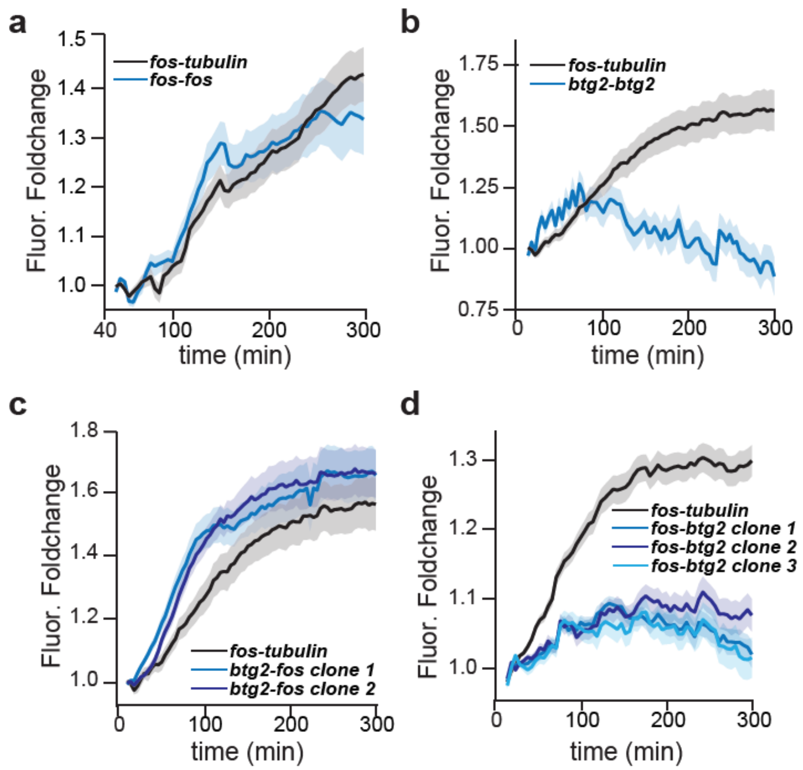

Supplementary Figure 7. The *BTG2* 3' UTR is necessary and sufficient for translation inhibition. (**A-D**) Quantification of YFP induction from clonal NIH3T3 cell lines expression each of four SynIEGs: *btg2-btg2* (in **A**), *fos-btg2* (in **B**), *btg2-fos* (in **C**), or *fos-fos* (in **D**). In each case, YFP induction was compared to a control *fos-tubulin* SynIEG. Means ± S.E.M are shown for at least 20 cells in each condition.

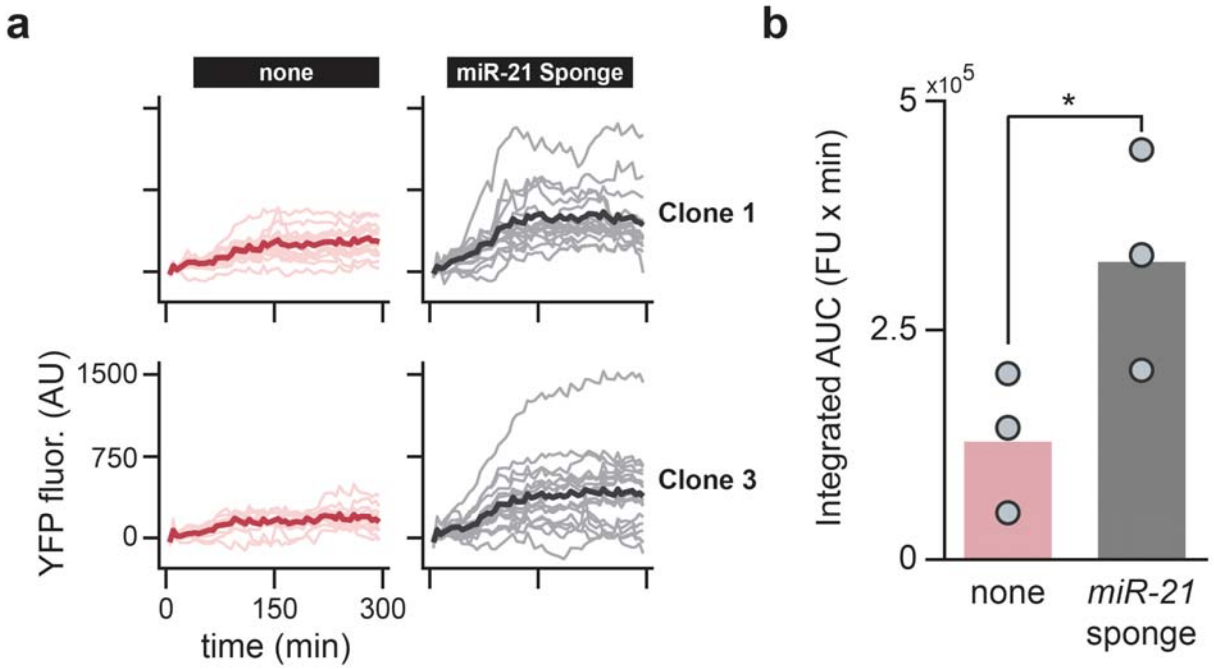

Supplementary Figure 8. Further replicates describing the effect miR-21 sponge on *BTG2* expression. **(A)** YFP induction traces for 2 separate clonal lines with (Clone 1: n = 15 cells; Clone 3: n = 19 cells) and without (Clone 1: n = 16 cells; Clone 3: n=10 cells) miR-21 sponge expression. Cells were treated with 10% serum after being in growth-free media for 5 hours. Gray traces are single cells and dark black line is the mean. **(B)** Quantification of the area-under-the-curve (AUC) of YFP induction after serum stimulation for the clonal SynIEG cell lines shown in Figure 3H and Supplementary Figure 9A (see Methods for quantification details). Each point represents the average of 20-30 cells from an independent clone. Statistics are derived using a paired two-sided t test (*p<0.05).

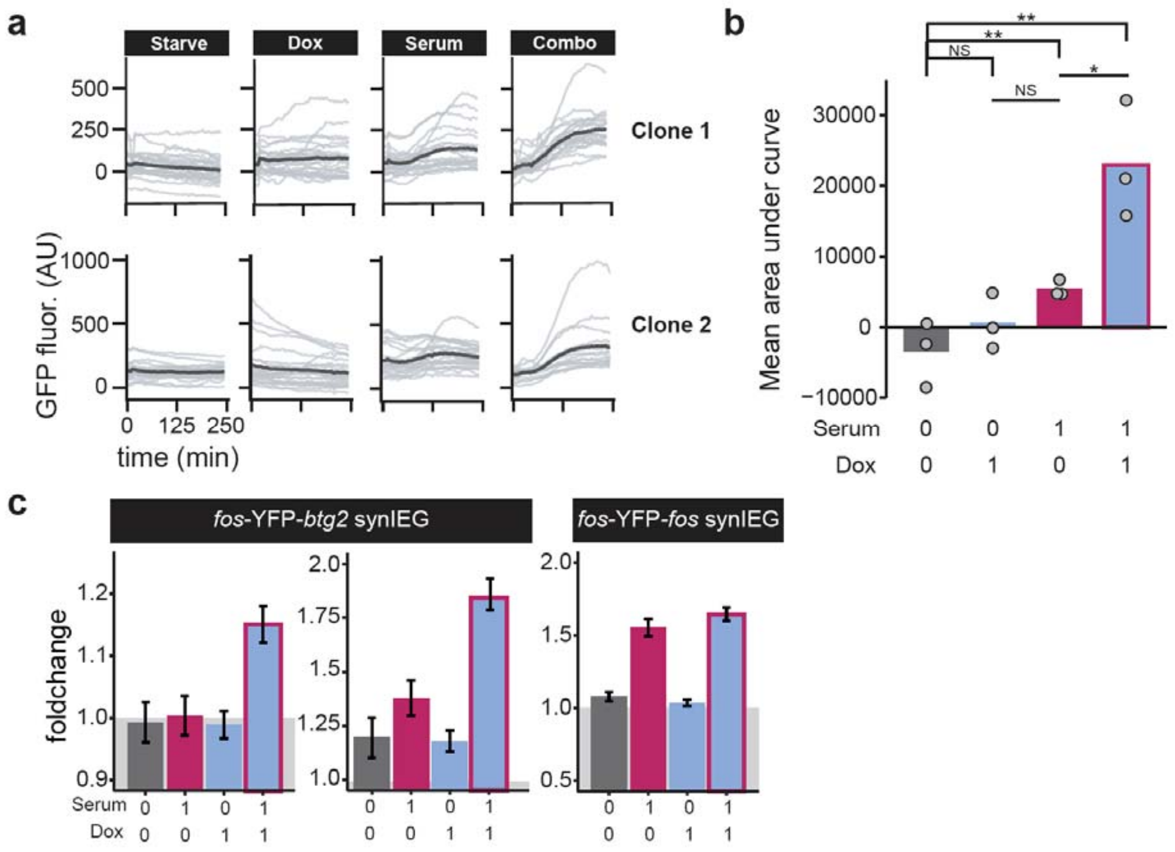

Supplementary Figure 9. Further characterization of the *fos-btg2* AND gate. (**A**) GFP induction traces for clonal NIH3T3 cells harboring the *fos*-dGFP-*btg2* SynIEG. Cells were treated with growth factor free media, 860 nM doxorubicin (Dox), 1% serum, or a combination of doxorubicin and serum. Gray traces are single cells and dark black line is the mean. (**B**) Quantification of ~20 cells for each condition in 3 biological replicates for clone 2 (see Methods). Each point is the mean of at least 20 cells in a single experimental replicate; the bar represents the mean of three repeats. Statistics are derived using an unpaired two-sided t test (*$p<0.05$, **$p < 0.01$, ***$p < 0.001$). (**C**) Fold-change in YFP induction for clonal NIH3T3 cell lines harboring either the *fos*-YFP-*btg2* SynIEG (2 independent clones; left panels) or the *fos*-YFP-*fos* SynIEG (right panel). Cells were treated with starvation media, 1% serum, 860 nM doxorubicin, or a combination of doxorubicin and serum. Bar height represents the mean YFP fold-change over initial time-point after 300 minutes of stimulation and error bars represent S.E.M for at least 20 cells per condition.

Supplementary Table 1: Resources and Plasmids

REAGENT or RESOURCE	SOURCE	IDENTIFIER
**Bacterial and Virus Strains**		
Stellar Chemically Competent Cells	Clontech Laboratories	Cat# 636763
**Chemicals, Peptides, and Recombinant Proteins**		
CloneAmp HiFi PCR Polymerase	Clontech Laboratories	Cat#639298
Cycloheximide	Sigma-Aldrich	Cat# C104450
Doxorubicin	Fischer Scientific	Cat# ICN15910101
Trichostatin A	Tocris	Cat# 1406
Fugene HD	Promega	Cat# E2311
Lipofectamine LTX with Plus Reagent	Thermo-Fischer	Cat# 15338100
PrimeSTAR GXL DNA Polymerase	Clontech Laboratories	Cat# R050B
QuickExtract DNA Extraction	Epicenter Bio	Cat# QE09050
**Experimental Models: Cell Lines**		
NIH 3T3 Cells	ATCC	Cat# CRL-1658
Lenti-X 293t Cells	Clontech Laboratoires	Cat# 632180
‘‘Chassis’’ cell line (NIH 3T3 + optoSOS + H2B-diRFP + MCP-mCherry)	Wilson et. al., 2017; Ref. [1]	Available upon request
**Recombinant DNA**		
pCMV-dR8.91 lenti helper plasmid	Gift from the Trono lab	Available upon request
pMD2.G lenti helper plasmid	Gift from the Trono lab	Addgene #12259
pHR *fos-tubulin* SynIEG	This paper	Available upon request
PigBac *fos-tubulin* SynIEG	This paper	Available upon request
PigBac *fos-fos* SynIEG	This paper	Available upon request
PigBac *fos-btg2* SynIEG	This paper	Available upon request
PigBac *btg2-fos* SynIEG	This paper	Available upon request
PigBac *btg2-btg2* SynIEG CMV BFP	This paper	Available upon request
PigBac *fos*-dGFP-*btg2* SynIEG CMV BFP	This paper	Available upon request
PigBac *fos*-YFP-Fra1-*fos* SynIEG	This paper	Available upon request
pHR U6 “*empty*” CMV BFP-NLS	This paper	Available upon request
pHR U6 miR-21 CMV BFP-NLS	This paper	Available upon request
pHR U6 miR-21 inhibitor CMV BFP-NLS	This paper	Available upon request
pHR BFP-SSPB-SOScat p2a iLID-CAAX	Ref. [2]	Available upon request (Addgene number forthcoming)
pLenti TOPFLASH d2EGFP	Gift from Reya lab; Ref. [3]	Addgene #14715

Supplementary Note 1. Sequences of important UTRs and degrons used in the manuscript

1.1 *FOS* 5' UTR sequence

cagcgagcaactgagaagactggatagagccggcggttccgcgaacgagcagtgaccgcgctcccacccagctctgc
tctgcagctcccaccagtgtctacccctggaccccttgccgggctttccccaaacttcgac

1.2 *FOS* 3' UTR sequence

gcagtcagagaaggcaaggcagccggcatccagacgtgccactgcccgagctggtgcattacagagaggagaaacac
gtcttccctcgaaggttcccgtcgacctagggaggaccttacctgttcgtgaaacacaccaggctgtgggcctcaag
gacttgcaagcatccacatctggcctccagtcctcacctcttccagagatgtagcaaaaacaaaacaaaacaaaaca
aaaaaccgcatggagtgtgttgttcctagtgacacctgagagctggtagttagtagagcatgtgagtcaaggcctgg
tctgtgtctcttttctctttctccttagttttctcatagcactaactaatctgttgggttcattattggaattaacc
tggtgctggattgtatctagtgcagctgattttaacaatacctactgtgttcctggcaatagcgtgttccaattaga
aacgaccaatattaaactaagaaaagataggactttattttccagtagatagaaatcaatagctatatccatgtact
gtagtccttcagcgtcaatgttcattgtcatgttactgatcatgcattgtcgaggtggtctgaatgttctgacatta
acagtttttccatgaaaacgtttttattgtgttttcaatttatttattaagatggattctcagatatttatattttta
ttttatttttttctaccctgaggtctttcgacatgtggaaagtgaatttgaatgaaaaattttaagcattgtttgct
tattgttccaagacattgtcaataaaagcatttaagttgaa

1.3 *BTG2* 5' UTR sequence

gagtggtatgaaaggcgcagcccggggaaagtccgggcagagcccgagaggtggccagaccgtcatcatcgttctaa
tacagctacttcctcagccaccggt

1.4 *BTG2* 3' UTR sequence

ataggagccacccgaccctggcactctactgttctcatgctgccctgacaacaggccaccgtatacctcaacctggg
gaactgtatttttaaaatgaagagctatttatacatgttttttttttttttttaagaaaagaggaaaaaaaccaa
aagattttttttaaaaaaaaaagaaaaagaaaaacaattctttaaagggagctgcttggaagtggcctccccaggtg
cctttggagagaactgttcttgattgcatctgtgagccagtgtttgcctaggggaatgggttggggattggcctagc
caaggtaaaaggggattcttggctgatccccccaggaggtggtggaagggagcaaggttagcaactgtgaatgagag
gggtcagggtctgctctgggttaccgtcccagctgggatgcctgtatgcctggtccctctcttactcaggggcattc
aagcctgatcttaaataatactacattgcctaatcttctcttttgtttttcggctgagatcaggggcagactgaaag
gcctctcctgtcccttctgttctaagcagtctcttgaagccgtgtctcgtttctgagtctacccttgggggcctgaa
gagcttgcttcccagcccggaatctgtctaaacatttttggagggtgggatgtaaggcaggcgcgcaaagactttg
gggctaagatggagaggacctgcacaaaacctttgctttgctctgtgctgctttgtatgggtggatagtgaataatt
tagggatgatttgcaatggaattttgggacccaaagagtatccaatgggggtaggtcttttggacccagtccttcct
tttgggaaccacacgacagtctgaatgctgctaccactattcctttgagaggtggctcaaagctccagggaactcca
agtcctttcttactgccttctcttcaagagcaaccttccccatttttctttcccctttcctgcggttgggtcctgg
agggccctatttcctaggacaagcgttctcagtcactgtgcaatagtcccaggatgctctgagaccggacctcccag
cccctcctgatgccctggtaggttttagggacccattcttcccattcttctaggggttctgactggctggtgggcct
tgcggagatcttcctgggccacagggagggcacctgtgcactgcaggactacctggtattcttgtagggctgccatg

aagagtcaaaccttgggcacagctttagctccttggtgctcagagcacctgtgggggaggttacctctctctctc
ttagtaaaatccaaatttatttgtagatgtgtgcaatatttactgttctgggttggagaaaattgggaaacactggg
aagaaatggctttccttcaggttcagtgacactgatgagggcttcttagaaggcctcaagtctctcaaactgaagga
cagagctagagctagcccgtcacccсttggtgaggattcccttccccgtttctctccaccgccatggcatcctgtgt
cctagatttctcagctcctcagtttctgctcaaaggtgctatttaccaaactctctgcctgcccgggcagacaggcc
ccagcttcgcacagccttcccaggtggcttcgtctctcttgctttaaccttaactctgggcccacagacctgagagc
tgtggcctacaacaaagctgtgaattgttccagatggttcttgtgttttgtccgcacacaggtgcctgccgtttaga
agctgcctcctggtctcatgcttaaatcttcaattctttactgtccttgttcactttagaaatgacaaaccctagag
ctggactgttgagcaggcctgtctctcttattaagtaaaaataagaaatagtggtaagtttgtaagctattctgaca
gaaaagacaaaggttactaattgtataatagcgcttttatatggaagactgtacagctttatggacaaatgtaaact
ttcttttttgtttttaataaaaatgtagcagatcgtgtaatgtgtagagaaggtggaattccatagcgctgactggc
cctcttagatacaaaccccttcgtgtttcgtggacttcatcagttgtcctgagctccgtctgcagcatgcagacgtt
ataaaagataccctaggtttgtgacgaggttgcttccattgcatcccctctccttgggaggcggctccatacttctg
tccttgtgaatattgtcacaggtctcttaaaaaacaaacaaacaaaaaagcccaaaaaccaatatgttatctgcaca
ctctgcagccagttccatagttttgctcttggccattcagcacattgaagagctggccagctgtgtccacatctgcg
caagcaagccaccacggctggaaactatataaagaaaagaaaccatccagaaagttccaaagacacaagacttgggt
ttggcacacactaagcacaccagtgcactcgatgcgactg

1.5 *TUBULIN* 3’ UTR sequence

ttcttaaagcttttactttgagacatcatggaaaacttaagaggtacaacatggagaagacatgatcacagaatgga
aacagcacagaagcatcagtgcctgcaactaatactggagcagtttgacgacacagggggctcaaggaatggactta
gtactcctctccttcttcctccccctcgccttccacagaatccacaccaacctcctcataatccttctctagggcag
ccatgtcctcacgggcctcagagaactcaccctcctccatgccctcacccacataccagtgcacaaaggcacgcttg
gcatacatcagatcaaacttgtgatctaggcgagcccaggcctcagcaatggctgtggtgttgctcagcatgcacac
agctctctgcaccttggccaggtcaccaccgggtaccacagtgggaggctggtaattaatgccaaccttgaagccag
tggggcaccagtctacaaactggatgctgcgcttggtcttgatggtggcaatggcagcattgacatctttgggaacc
acatcaccacggtatagcaggcagcaagccatgtatttaccatggcgagggtcacatttcaccatctggttggctgg
ctcaaagcaggcattggtgatctctgctacagaaagctgctcatggtaggctttctcagcagagatgacaggggcat
aagtggccagagggaagtggatgcgagggtagggtaccaggttggtctggaattctgtcagatcaacattcagggcc
ccatcaaatctgagggaagcagtgatggaagacacaatctggctaataaggcggttaaggttggtgtaggttgggcg
ctcaatgtcgaggtttctacgacagatgtcatagatggcctcattgtctaccatgaaggcacaatcagagtgctcca
gggtggtgtgggtggtgaggatggaattgtagggctcaaccacagcagtggaaacctgggggctgggtaaatggag
aactccagcttggacttctttccgtaatccacagagagccgctccatcagcagggaggtgaagccagagccagttcc
cccaccaaagctgtggaaaaccaagaagccctggagacctgtgcactggtcagc

1.6 Fra1 degron amino acid sequence

LVLEAHRPICKIPEGDKKDPGGSGSTSGASSPPAPGRPVPCISLSPGPVLEPEALHTPTLMTTPSLTPFTPSLVFTY
PSTPEPCSSAHRKSSSSSGDPSSDPLGSPTLLAL

Supplementary References

1. Wilson, M.Z., Ravindran, P.T., Lim, W.A., and Toettcher, J.E. (2017). Tracing Information Flow from Erk to Target Gene Induction Reveals Mechanisms of Dynamic and Combinatorial Control. Molecular cell *67*, 757-769 e755.
2. Goglia, A.G., Wilson, M.Z., Jena, S.G., Silbert, J., Basta, L.P., Devenport, D., and Toettcher, J.E. (2020). A Live-Cell Screen for Altered Erk Dynamics Reveals Principles of Proliferative Control. Cell Syst *10*, 240-253 e246.
3. Reya, T., Duncan, A.W., Ailles, L., Domen, J., Scherer, D.C., Willert, K., Hintz, L., Nusse, R., and Weissman, I.L. (2003). A role for Wnt signalling in self-renewal of haematopoietic stem cells. Nature *423*, 409-414.
