# Supplementary material for: Engineering combinatorial and dynamic decoders using synthetic immediate-early genes
Source: Commun Biol. 2020 Aug 13;3:436. doi: 10.1038/s42003-020-01171-1 (PMC7426417; doi:10.1038/s42003-020-01171-1)
Supplement: Supplementary file 2 — Description of Additional Supplementary Files [file 42003_2020_1171_MOESM2_ESM.pdf]

## Description of Additional Supplementary Files

**File Name**: **Supplementary Movie 1**
**Description**: Time-lapse imaging of NIH 3T3 containing fos-btg2 SynIEG in the MCP mCherry channel to visualize transcriptional induction. MCP-mCherry images were collected at 0.7 µm z-stacks and the processed using maximum intensity projection. Cells were stimulated with serum and images were acquired every 3 minutes

**File Name**: **Supplementary Data 1**
**Description**: Raw data for all experiments

**File Name**: **Supplementary Data 2**
**Description**: MATLAB scripts to analyze bursts. Included are the analysis script as well as test data sets to recapitulate figures from the paper

**File Name**: **Supplementary Data 3**
**Description**: R scripts to analyze protein fluorescence. Included are the analysis script and test data sets
