# Supplementary figures and images for: Engineering combinatorial and dynamic decoders using synthetic immediate-early genes
Source: Commun Biol. 2020 Aug 13;3:436. doi: 10.1038/s42003-020-01171-1 (PMC7426417; doi:10.1038/s42003-020-01171-1)

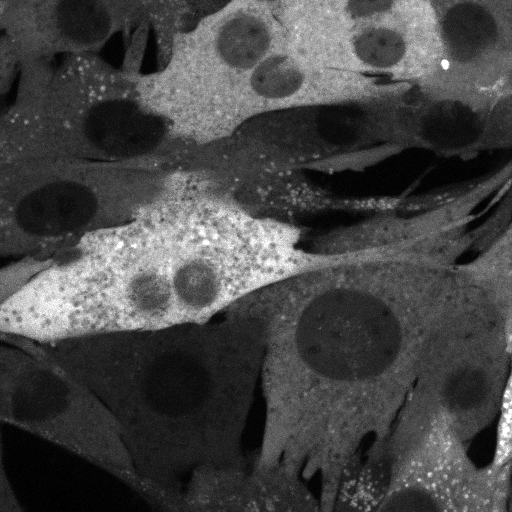

Supplement: Supplementary file 4 — Supplementary Data 2 [file 42003_2020_1171_MOESM4_ESM.zip › Dataset/V_BandPass001-MaxIP.nd2 - Z=5 C=0.tif]
