# Supplementary material for: Engineering combinatorial and dynamic decoders using synthetic immediate-early genes
Source: Commun Biol. 2020 Aug 13;3:436. doi: 10.1038/s42003-020-01171-1 (PMC7426417; doi:10.1038/s42003-020-01171-1)
Supplement: Supplementary file 7 — Reporting Summary [file 42003_2020_1171_MOESM7_ESM.pdf]

nature research

Corresponding author(s): Jared E. Toettcher

Last updated by author(s): Jun 10, 2020

Reporting Summary

Nature Research wishes to improve the reproducibility of the work that we publish. This form provides structure for consistency and transparency in reporting. For further information on Nature Research policies, see Authors & Referees and the Editorial Policy Checklist.

Statistics

For all statistical analyses, confirm that the following items are present in the figure legend, table legend, main text, or Methods section.

n/a	Confirmed	
☐	☒	The exact sample size (*n*) for each experimental group/condition, given as a discrete number and unit of measurement
☐	☒	A statement on whether measurements were taken from distinct samples or whether the same sample was measured repeatedly
☐	☒	The statistical test(s) used AND whether they are one- or two-sided<br>*Only common tests should be described solely by name; describe more complex techniques in the Methods section.*
☒	☐	A description of all covariates tested
☐	☒	A description of any assumptions or corrections, such as tests of normality and adjustment for multiple comparisons
☐	☒	A full description of the statistical parameters including central tendency (e.g. means) or other basic estimates (e.g. regression coefficient) AND variation (e.g. standard deviation) or associated estimates of uncertainty (e.g. confidence intervals)
☒	☐	For null hypothesis testing, the test statistic (e.g. *F*, *t*, *r*) with confidence intervals, effect sizes, degrees of freedom and *P* value noted<br>*Give P values as exact values whenever suitable.*
☒	☐	For Bayesian analysis, information on the choice of priors and Markov chain Monte Carlo settings
☒	☐	For hierarchical and complex designs, identification of the appropriate level for tests and full reporting of outcomes
☒	☐	Estimates of effect sizes (e.g. Cohen's *d*, Pearson's *r*), indicating how they were calculated

Our web collection on statistics for biologists contains articles on many of the points above.

Software and code

Policy information about availability of computer code

Data collection	All imaging data was acquired using Nikon NIS-Elements Version 4.4 software.
Data analysis	All of the custom code written in R (change in cell fluorescence) and in MatLAB (bursting analysis) have been supplied with example datasets as supplementary files.

For manuscripts utilizing custom algorithms or software that are central to the research but not yet described in published literature, software must be made available to editors/reviewers. We strongly encourage code deposition in a community repository (e.g. GitHub). See the Nature Research guidelines for submitting code & software for further information.

Data

Policy information about availability of data

All manuscripts must include a data availability statement. This statement should provide the following information, where applicable:

- Accession codes, unique identifiers, or web links for publicly available datasets
- A list of figures that have associated raw data
- A description of any restrictions on data availability

Raw data for all experiments have been included as a supplementary data file. Additional data for experiments are available upon reasonable request.

Field-specific reporting

Please select the one below that is the best fit for your research. If you are not sure, read the appropriate sections before making your selection.

☒ Life sciences ☐ Behavioural & social sciences ☐ Ecological, evolutionary & environmental sciences

October 2018

Life sciences study design

All studies must disclose on these points even when the disclosure is negative.

Sample size	No statistical sample size calculations were performed, but adequate samples and replicates were analyzed in each experiment to ensure reproducibility of results.
Data exclusions	No data were excluded.
Replication	All imaging data was acquired and reproduced multiple times.
Randomization	No randomization was performed.
Blinding	No blinding was performed.

Reporting for specific materials, systems and methods

We require information from authors about some types of materials, experimental systems and methods used in many studies. Here, indicate whether each material, system or method listed is relevant to your study. If you are not sure if a list item applies to your research, read the appropriate section before selecting a response.

Materials & experimental systems

n/a	Involved in the study	
☒	☐	Antibodies
☐	☒	Eukaryotic cell lines
☒	☐	Palaeontology
☒	☐	Animals and other organisms
☒	☐	Human research participants
☒	☐	Clinical data

Methods

n/a	Involved in the study	
☒	☐	ChIP-seq
☒	☐	Flow cytometry
☒	☐	MRI-based neuroimaging

Eukaryotic cell lines

Policy information about cell lines

Cell line source(s)	NIH3T3 cells, HEK293T cells were purchased from ATCC and Takara Bio, respectively.
Authentication	Cell lines were not authenticated beyond what was performed by the original providers.
Mycoplasma contamination	Cell lines are periodically tested for mycoplasma and only negative-testing cell lines are used in the lab.
Commonly misidentified lines (See ICLAC register)	No commonly misidentified cell lines were used in this study.
